# Profile of Patients with Novel Coronavirus Disease 2019 (COVID-19) in Osaka Prefecture, Japan: A Population-Based Descriptive Study

**DOI:** 10.3390/jcm9092925

**Published:** 2020-09-10

**Authors:** Taro Takeuchi, Tomoka Imanaka, Yusuke Katayama, Tetsuhisa Kitamura, Tomotaka Sobue, Takeshi Shimazu

**Affiliations:** 1 Department of Social Medicine, Osaka University Graduate School of Medicine, Suita, Osaka 565-0871, Japan; tarogauss106072@gmail.com (T.T.); lucky_unatan@yahoo.co.jp (T.K.); tsobue@envi.med.osaka-u.ac.jp (T.S.); 2 Department of Traumatology and Acute Critical Medicine, Osaka University Graduate School of Medicine, Suita, Osaka 565-0871, Japan; rakimarupappara@yahoo.co.jp (T.I.); shimazu@hp-emerg.med.osaka-u.ac.jp (T.S.)

**Keywords:** COVID-19, elderly, PCR testing, epidemiology, Japan

## Abstract

Little is known about the epidemiological characteristics of patients with coronavirus disease 2019 (COVID-19) in Japan. This is a retrospective observational study of COVID-19 patients; study was conducted from February 1 to May 31, 2020. We used publicly collected data on cases of COVID-19 confirmed by polymerase chain reaction (PCR) testing in Osaka Prefecture, Japan. We described the patient characteristics. The Cox proportional-hazards model was applied to evaluate the association between factors (sex, onset month, age group, city of residence) and mortality, and hazard ratios (HRs) with 95% confidence intervals were estimated. During the study period, 5.7% (1782/31,152) of individuals who underwent PCR testing for COVID-19 showed positive results. Among 244 patients with information on symptoms, the most common symptom was fever (76.6%), followed by cough (44.3%). Of the 1782 patients, 86 patients died. Compared with those aged 0–59 years, higher mortality was observed among those aged 60–69 years (HR: 12.02 [3.37–42.93]), 70–79 years (HR: 44.62 [15.16–131.30]), 80–89 years (HR: 68.38 [22.93–203.89]), and ≥90 years (HR: 144.71 [42.55–492.15]). In conclusion, in Osaka Prefecture, Japan, the most common symptom was fever, and older adults had higher mortality among COVID-19 patients.

## 1. Introduction

The coronavirus disease 2019 (COVID-19) was identified in Wuhan, China in December 2019, and the COVID-19 outbreak has spread not only to China, but also to other countries all over the world. The outbreak of COVID-19 has been expanding worldwide [1]. In Japan, the number of patients with COVID-19 on May 31 was 16,851 [2]. The common symptoms of COVID-19 are fever, cough, sore throat, and general malaise, but some patients with COVID-19 are asymptomatic [3]. In contrast, some COVID-19 patients with severe condition, which account for about 20% of infected individuals [4], require intensive care, such as mechanical ventilation and extracorporeal membrane oxygenation. However, little is known about the age, sex, transmission route, or outcome of patients with COVID-19 in Japan.

Osaka Prefecture is the largest metropolitan area in western Japan, with a population of 8.8 million and a total area of 1905 km^2^. The number of patients with COVID-19 in Osaka Prefecture was about 1800 at the end of May, 2020 [2]. It has the second highest number of patients with COVID-19 in Japan after Tokyo [2]. This study aimed to determine the fundamental characteristics of patients with COVID-19 in Osaka Prefecture.

## 2. Methods

### 2.1. Study Design and Settings

This study was a retrospective observational study, and was conducted from 1 February to 31 May 2020. We used publicly collected data on cases of COVID-19 confirmed by polymerase chain reaction (PCR) testing in Osaka Prefecture, Japan [5]. As we used anonymous data, the necessity of obtaining informed consent from the participants was waived. This study was approved by the Ethics Committee of Osaka University Graduate School of Medicine (approval no. 20089).

### 2.2. Official Data Collection of COVID-19 Cases in Osaka Prefecture

In Osaka Prefecture, those suspected of having COVID-19 based on their medical history and travel were transferred to a hospital that specializes in the management of COVID-19 for PCR testing. In cases were a COVID-19 outbreak has been reported in places such as bars and live music venues, the staff in each public health center in charge followed up on the people involved, and data on the individuals with positive PCR test results were collected to identify if they were asymptomatic or not. All patients who showed positive PCR test results for COVID-19 were reported to the public health centers in accordance with the Infectious Disease Control Law [6]. Based on the reported patient information, the staff in each public health center collected further information including symptoms and onset dates. The onset date was defined as the date when any symptoms appeared (for patients with symptoms) and the date when the patient came into close contact with other individuals with COVID-19 (for patients with no symptoms). Detailed information on the response to the COVID-19 outbreak in Osaka Prefecture is available on the website of Osaka Prefecture [7].

### 2.3. Statistical Analyses

In this study, we describe the features of COVID-19 patients in Osaka Prefecture, and analyze their mortality by survival analyses. A total of 244 COVID-19 patients who showed a positive PCR test result between 1 February, 2020 and 31 March, 2020 were included. Detailed information about the patients with COVID-19 up to 31 March, 2020 is available on the website of Osaka Prefecture [5]. Based on X-ray or CT scan images, we divided the features into fever, cough, headache, sore throat, general malaise, mucus/nasal obstruction, low back pain, arthralgia, diarrhea, shortness of breath, and pneumonia. As for the analysis of mortality, 1782 COVID-19 patients who showed a positive PCR test result between 1 February, 2020 and 31 May, 2020 were included. All patients were followed for a maximum of 40 days from the onset date. The median number of days from the onset date to a positive PCR test result was summarized according to the onset month (February, March, April, and May) and city of residence (Osaka City, other cities, or unknown); these number of days were compared between each group using a Kruskal-Wallis test. A total of 86 COVID-19 patients who died were followed until the date of death, but two patients lacked data on the date of death during the study period. The characteristics of patients included in the analysis of mortality were summarized according to age at onset date, the onset month, and sex. The Kaplan-Meier method was applied to estimate the survival function according to (A) sex, (B) the onset month (February, March, April, and May), and (C) age group at the onset date (0–59, 60–69, 70–79, 80–89, and ≥90 years). Patients with missing information for the onset date or date of death were excluded from the analysis. We could not include patients with missing information on date of death as censored observations, because we did not have information on their date of latest confirmation on survival. A log-rank test was conducted to compare the mortality between each group. Based on the findings of previous studies, we assumed that sex [8], time of onset [9], age [10,11,12,13], and city of residence [14] would be associated with mortality. Therefore, the Cox proportional-hazards model was applied to evaluate the association of these factors with mortality among COVID-19 patients, and hazard ratios (HRs) with 95% confidence intervals (CIs) were estimated. Patients with missing information on the onset date or date of death were also excluded. Based on the available public information on the website of Osaka Prefecture, sex (male/female), onset month (February/March/April/May), age group (0–59, 60–69, 70–79, 80–89, ≥90 or unknown), and city of residence (Osaka City, other cities, or unknown) were mutually adjusted. All analyses were conducted using the STATA version 16.0 MP software (StataCorp. LP (4905 Lakeway Drive College Station, Texas 77845 USA)). All tests were two tailed, and *p*-values < 0.05 were considered significant.

## 3. Results

The number of PCR tests for COVID-19 in Osaka Prefecture was 31,152 from 1 February, 2020 to 31 May, 2020, and 1782 (5.7%) patients tested positive for COVID-19.

### 3.1. The Description of Features

A total 244 of patients from Osaka Prefecture were reported to have COVID-19 as of March 31, 2020. Table 1 shows the distribution of features by age group and sex. The most common feature was fever (187 patients, 76.6%), followed by cough (108 patients, 44.3%) and pneumonia (87 patients, 35.7%). The largest group was those aged 40–49 years (63 patients, 25.8%), followed by those aged 20–29 years (47 patients, 19.3%) and 50–59 years (39 patients, 16.0%). A total of 125 (51.2%) patients were men, while 119 (48.8%) were women. The most common feature among men was fever in 102 patients (81.6%), followed by cough in 60 patients (48.0%) and pneumonia in 55 patients (44.0%), whereas it was fever in 85 patients (71.4%), followed by cough in 48 patients (40.3%), general malaise in 32 patients (26.9%), and pneumonia images in 32 patients (26.9%) among women.

The median number of days from the onset date until a positive PCR test result according to the onset month was 11 days in February (interquartile range [IQR], 9–13), 8 days in March (IQR: 5–11), 6 days in April (IQR: 4–10), and 4 days in May (IQR: 3–7). The number of days was significantly different between the four groups (*p* < 0.001). The median number of days from the onset date to a positive PCR test result according to city of residence was 8 days in Osaka City (IQR: 5–11) and 7 days in other cities (IQR: 4–10), and the number of days was significantly different between the two areas (*p* < 0.001).

### 3.2. Mortality Analyses

Table 2 describes the distribution of age groups at the onset date according to the onset month. N (%) are described in this table. The total proportion of patients aged 70 years or over was 6% in February, 11% in March, 19% in April, and 31% in May; patients with later onset months were older than those with earlier onset months.

Table 3 describes the characteristics of the 1782 (men: 977, women: 805) patients included in the analysis of mortality during the study period. Among men and women, the percentage of deaths was higher in the older age group at the onset date. None of the male patients aged ≥90 years died, whereas 42% of the female patients aged ≥90 years died. A higher mortality rate was observed among patients with later onset months.

**Figure 1:**
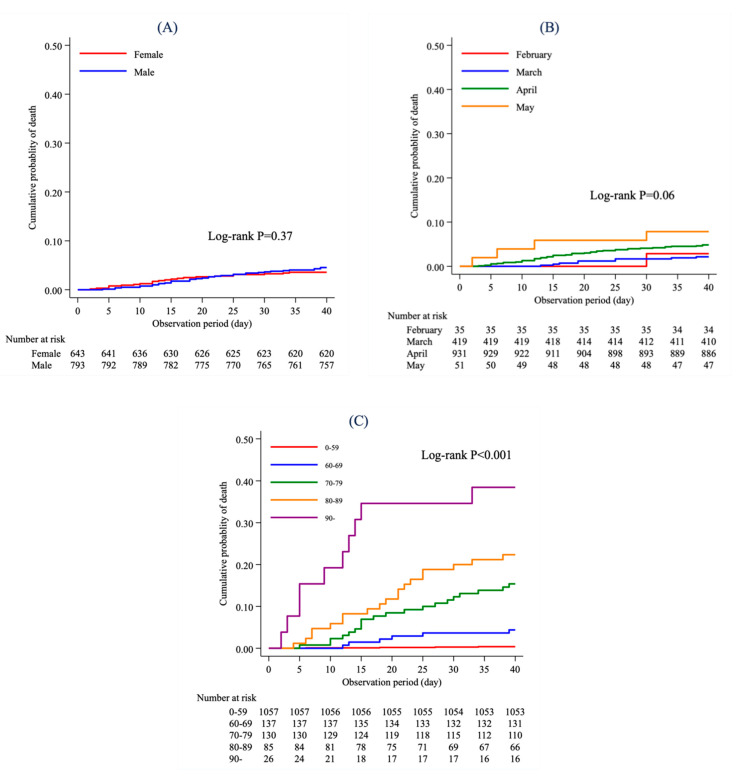
Kaplan-Meier curve according to (**A**) sex, (**B**) onset month, and (**C**) age group at the onset date.

Figure 1 shows the Kaplan-Meier curve for mortality according to (A) sex, (B) onset month, and C) age group at the onset date among COVID-19 patients in Osaka Prefecture. A total of 1436 patients for whom we obtained information on both the onset date and the date of death were included in this analysis. The log-rank test revealed that the difference in mortality between male and female was not statistically significant (*p* = 0.37), nor was the difference in mortality between onset month (*p* = 0.06). However, the difference in mortality between age groups at the onset date was statistically significant (*p* < 0.001). The cumulative mortality was highest in the group aged ≥90 years. Higher mortality was observed in the older age groups at the onset date.

Table 4 describes the results of Cox regression analysis among COVID-19 patients in Osaka Prefecture. Among the 1782 COVID-19 patients, 1436 for whom we obtained information on both the onset date and the date of death were included in this analysis. Of these, 59 patients died during the observation period. Mortality was similar between males and females, although the HR for men was greater than 1.0 (HR: 1.69, 95% CI: 0.95–3.02). The mortality rate was similar according to the onset month. Compared with patients aged 0–59 years at the onset date, mortality was higher among those aged 60–69 years (HR: 12.02, 95% CI: 3.37–42.93), 70–79 years (HR: 44.62, 95% CI: 15.16–131.30), aged 80–89 years (HR: 68.38, 95% CI: 22.93–203.89), and 90 years or over (HR: 144.71, 95% CI: 42.55–492.15). The mortality rate was higher in patients living in Osaka City than in those living in other cities (HR: 1.76, 95% CI: 1.02–3.04).

## 4. Discussion

This study revealed the characteristics and outcomes of patients with COVID-19 in Osaka Prefecture, Japan, between 1 February and 31 May, 2020. Fever was the most common feature of the COVID-19 patients, and the time interval from onset until a positive PCR test result decreased over time. This study also found that older age and residency in Osaka City were associated with higher mortality among the COVID-19 patients. This study provides useful information for the prevention and diagnosis of COVID-19 with the use of comprehensively collected data from Osaka Prefecture in Japan.

Some previous studies have reported the distribution of symptoms in COVID-19 patients [15,16,17]. In a meta-analysis of epidemiological studies on COVID-19 in China [15], 88.5% of the patients had fever, 68.6% had cough, 35.8% had muscle pain or malaise, and 28.2% had sputum. In a study of 463 patients in five metropolitan hospitals in Detroit, United States, the most common symptom was cough (74.9%), followed by fever (68.0%), dyspnea (60.9%), and myalgias (42.0%) [16]. In another study of 582 patients aged below 18 years at 82 hospitals in 25 European countries [17], the most common symptom was fever (65%), followed by upper airway symptoms (54%), headache (28%), and lower respiratory tract symptoms (25%). Thus, fever and cough were the most common symptoms in our study and in previous studies. In contrast, the percentage of patients with gastrointestinal symptoms was different between this study and previous studies. The proportion of patients who developed gastrointestinal symptoms such as vomiting and diarrhea was 20% in the United States [16] and European countries [17]; however, only a few patients with gastrointestinal symptoms were reported in a previous study in China [15] and in our study. The mechanism and reason for this difference is unknown, but it may be due to the racial differences in pathogenesis and/or differences in the types of viruses. Hence, further research is needed to clarify this point. In addition, a previous study revealed significantly higher rates of symptoms such as dyspnea, anorexia, nausea, and diarrhea in hospitalized patients compared with those discharged and sent home [16]. Most previous studies used hospital-based data, while our study used a population-based dataset collected in accordance with the Infectious Disease Control Law. This difference in the dataset used possibly led to the differences in the distribution of symptoms.

From February to May 2020, the time interval from onset until a positive PCR test result was shortened. To reduce the time interval from onset to testing, it is important that infected individuals visit the hospital as early as possible and undergo PCR testing as needed. To the best of our knowledge, although no previous studies have evaluated the time interval from onset to PCR testing in COVID-19 patients, the median time from onset to hospitalization is 4–5 days in other countries [2]. Compared with the time interval from onset to hospitalization in other countries, the time interval from onset to positive PCR test result in this study was relatively long, which was possibly influenced by the following two factors. In Japan, where the pandemic was not widespread between February and early March, infected individuals did not suspect that they had a COVID-19 infection; instead, they only regarded it as a common cold and did not visit the hospital immediately, even if they started developing symptoms. As the global outbreak of COVID-19 became known after late March, the public’s awareness of COVID-19 changed, and they began to visit the hospital early, which may have shortened the time interval between onset and PCR testing over time. Thus, when an outbreak of an emerging infectious disease is suspected, the public must be informed and encouraged to visit a hospital at an early stage to prevent the spread of infection. Another possible factor was that the PCR testing system for COVID-19 was not widely adopted in Japan compared with other countries. As a result, PCR testing for COVID-19 was conducted only in some specific laboratories, which may have resulted in longer testing times. By May 2020, PCR testing for COVID-19 patients had become more available, which possibly contributed to the shorter time between onset and positive PCR test results in Osaka Prefecture. The development of a system capable of performing tests to identify infectious pathogens, such as PCR testing, is also essential for the early recognition of an epidemic and to prevent the spread of infection.

In addition, a higher mortality was observed among patients living in Osaka City compared with those living in other cities. Although the reasons for this association remain unclear in this study, it could be explained in part by the difference in the time interval from the onset to a positive result confirmed by PCR testing. This time interval was significantly longer in Osaka City than in other cities, which might suggest that the condition of patients was more severe before they were diagnosed with COVID-19 and were admitted to the hospital.

Furthermore, higher mortality was also observed among men compared with women, although this result was not statistically significant. A previous study conducted in foreign counties reported higher mortality among men compared with women [8]. Such sex differences could be explained in part by the differences in the mechanism of severe acute respiratory syndrome coronavirus 2 entry into the cell between men and women [8] or the proportion of patients who had a history of smoking. Hence, further elaborate studies are needed to verify sex differences in epidemiology and outcomes among COVID-19 patients.

In the present study, higher mortality was observed in older age groups, which is consistent with previous studies on COVID-19 conducted in Western countries and China [10,11,12,13]. Previous studies reported that higher age was one of the most important factors leading to critical illness [10,11,12,13]. A retrospective cohort study in Wuhan, China [11], revealed that older age was significantly associated with in-hospital mortality (adjusted odds ratio: 1.10, 95% CI: 1.03–1.17, per year increase; *p* = 0.0043). A prospective cohort study in New York City, United States, [12] also revealed that older age was significantly associated with in-hospital mortality (adjusted hazard ratio: 1.31, 95% CI: 1.09–1.57 per 10-year increase in age). A multicenter cohort study in Michigan, United States, [13] also revealed that the mortality was significantly higher in the group aged >60 years than in the group aged <60 years (adjusted odds ratio: 1.93, 95% CI: 1.26–2.94). These studies proposed several hypotheses about the mechanism underlying the higher mortality among older COVID-19 patients [18,19,20]: greater number of comorbidities among older patients [18], correlation between older age and increased viral load in the upper respiratory tract [19], and defects in T cell and B cell function among older patients [20]. Considering these results and the findings of our study, emergency measures for the treatment and prevention of COVID-19 in older patients are urgently needed.

There are several strengths in the present study. First, we provided detailed, fundamental information on symptoms according to age group and sex among COVID-19 patients in Osaka Prefecture, Japan. Second, to our knowledge, this is the first study in Japan to investigate the association between factors and mortality among COVID-19 patients using multivariable analyses.

There are several limitations to the present study. First, we did not have information on the date when the patients tested negative for COVID-19 after hospitalization. Therefore, we hypothesized that all the patients were followed up at a maximum of 40 days from the onset date, but the observation period for patients who were alive could have been biased due to this assumption. Second, we did not have detailed information on COVID-19 patients who tested positive on 1 April, 2020 or later because this information was not available. Third, we could not analyze causes of deaths among COVID-19 patients, because this information was not publicly available in Osaka Prefecture, Japan. Fourth, we could not include two patients with missing information on date of death in the survival analysis, because we only had access to publicly-available information on COVID-19 patients in Osaka Prefecture, Japan, and did not have detailed information on these patients. Finally, we could not adjust for various factors such as in-hospital treatments, comorbidities, past history, and health status in the Cox regression analysis, because this information was not publicly available.

## 5. Conclusions

In Osaka Prefecture, Japan, among COVID-19 patients, the most common symptom was fever, and older adults had higher mortality. Further evaluations of the cause of death among COVID-19 patients are also needed.

## Figures and Tables

**Table 1:** Distribution of features according to age group and gender.

	Total	Features																					
		Fever		Cough		Headache		Sore Throat		General Malaise		Mucus/ Nasal obstruction/ Sneeze		Low Back Pain		Arthralgia		Diarrhea		Shortness of Breath		Pneumonia	
Total, n (%)	244	187	(76.6)	108	(44.3)	6	(2.5)	29	(11.9)	65	(26.6)	32	(13.1)	1	(0.4)	1	(0.4)	2	(0.8)	19	(7.8)	87	(35.7)
Age group, n (%)																							
0–9	4	1	(25.0)	0	(0)	0	(0)	0	(0)	0	(0)	2	(50.0)	0	(0)	0	(0)	0	(0)	0	(0)	0	(0)
10–19	3	1	(33.3)	1	(33.3)	0	(0)	0	(0)	0	(0)	0	(0)	0	(0)	0	(0)	0	(0)	0	(0)	0	(0)
20–29	47	35	(74.5)	24	(51.1)	1	(2.1)	8	(17.0)	14	(29.8)	11	(23.4)	0	(0)	0	(0)	0	(0)	3	(6.4)	13	(27.7)
30–39	38	22	(57.9)	13	(34.2)	1	(2.6)	2	(5.3)	7	(18.4)	6	(15.8)	0	(0)	0	(0)	0	(0)	2	(5.3)	11	(29.0)
40–49	63	52	(82.5)	34	(54.0)	2	(3.2)	10	(15.9)	13	(20.6)	6	(9.5)	1	(1.6)	0	(0)	0	(0)	4	(6.4)	24	(38.1)
50–59	39	30	(76.9)	18	(46.2)	1	(2.6)	6	(15.4)	15	(38.5)	4	(10.3)	0	(0)	1	(2.6)	1	(2.6)	4	(10.3)	14	(35.9)
60–69	26	23	(88.5)	10	(38.5)	1	(3.9)	2	(7.7)	9	(34.6)	1	(3.9)	0	(0)	0	(0)	0	(0)	2	(7.7)	12	(46.2)
70–79	18	17	(94.4)	4	(22.2)	0	(0)	1	(5.6)	5	(27.8)	2	(11.1)	0	(0)	0	(0)	1	(5.6)	1	(5.6)	7	(38.9)
≥80	6	6	(100.0)	4	(66.7)	0	(0)	0	(0)	2	(33.3)	0	(0)	0	(0)	0	(0)	0	(0)	3	(50.0)	6	(100.0)
Gender, n (%)																							
Male	125	102	(81.6)	60	(48.0)	4	(3.2)	10	(8.0)	33	(26.4)	10	(8.0)	0	(0)	1	(0.8)	2	(1.6)	10	(8.0)	55	(44.0)
Female	119	85	(71.4)	48	(40.3)	2	(1.7)	19	(16.0)	32	(26.9)	22	(18.5)	1	(0.8)	0	(0)	0	(0)	9	(7.6)	32	(26.9)

**Table 2:** Distribution of age group at the onset date according to the onset month.

		Onset Month				
		February	March	April	May	Unknown
Age at Onset	0–59	27 (77.1)	345 (82.3)	655 (70.4)	30 (57.7)	236 (68.4)
	60–69	6 (17.1)	28 (6.7)	97 (10.4)	6 (11.5)	24 (7.0)
	70–79	2 (5.7)	29 (6.9)	94 (10.1)	5 (9.6)	46 (13.3)
	80–89	0 (0.0)	17 (4.1)	60 (6.4)	8 (15.4)	33 (9.6)
	≥90	0 (0.0)	0 (0.0)	24 (2.6)	3 (5.8)	6 (1.7)
	Unknown	0 (0.0)	0 (0.0)	1 (0.1)	0 (0.0)	0 (0.0)
Total		35	419	931	52	345

**Table 3:** Characteristics of patients included in the analysis of mortality.

	Male (N = 977)			Female (N = 805)		
	Number of Patients	Number of Deaths	Percentage of Death	Number of Patients	Number of Deaths	Percentage of Death
Age at onset						
0–59	701	5	0.7	592	0	0.0
60–69	90	8	8.9	71	1	1.4
70–79	108	21	19.4	68	9	13.2
80–89	71	19	26.8	47	12	25.5
≥90	7	0	0.0	26	11	42.3
Unknown	0	0		1	0	0.0
Onset month						
February	14	1	7.1	21	0	0.0
March	256	7	2.7	163	4	2.5
April	502	31	6.2	429	19	4.4
May	21	2	9.5	31	3	9.7
Unknown	184	12	6.5	161	7	4.3

**Table 4:** Results of Cox regression analysis in the present population.

	Number of Patients	Number of Deaths	HR (95% CI)
Sex			
Female	643	23	Ref
Male	793	36	1.69 (0.95–3.02)
Onset month			
February	35	1	2.06 (0.21–20.00)
March	419	9	1.03 (0.29–3.59)
April	931	45	1.15 (0.39–3.39)
May	51	4	Ref
Age group at onset			
0–59	1057	4	Ref
60–69	137	6	12.02 (3.37–42.93)
70–79	130	20	44.62 (15.16–131.30)
80–89	85	19	68.38 (22.93–203.89)
≥90	26	10	144.71 (42.55–492.15)
Unknown	1	0	Unconverged
Residence			
Other cities	793	26	Ref
Osaka City	604	27	1.76 (1.02–3.04)
Unknown	39	6	3.73 (1.33–10.45)

HR, hazard ratio; CI, confidence interval.
